# The CoREST complex regulates multiple histone modifications temporal-specifically in clock neurons

**DOI:** 10.1098/rsob.230355

**Published:** 2024-07-10

**Authors:** Pengfei Lv, Zhangwu Zhao, Yukinori Hirano, Juan Du

**Affiliations:** ^1^ Department of Entomology and MOA Key Lab of Pest Monitoring and Green Management, College of Plant Protection, China Agricultural University, Beijing 100193, People's Republic of China; ^2^ Division of Life Science, The Hong Kong University of Science and Technology, Clear Water Bay, Kowloon, Hong Kong

**Keywords:** CoREST, *Period*, circadian rhythm, histone modification, *Drosophila*

## Abstract

Epigenetic regulation is important for circadian rhythm. In previous studies, multiple histone modifications were found at the *Period* (*Per*) locus. However, most of these studies were not conducted in clock neurons. In our screen, we found that a *CoREST* mutation resulted in defects in circadian rhythm by affecting *Per* transcription. Based on previous studies, we hypothesized that *CoREST* regulates circadian rhythm by regulating multiple histone modifiers at the *Per* locus. Genetic and physical interaction experiments supported these regulatory relationships. Moreover, through tissue-specific chromatin immunoprecipitation assays in clock neurons, we found that the *CoREST* mutation led to time-dependent changes in corresponding histone modifications at the *Per* locus. Finally, we proposed a model indicating the role of the CoREST complex in the regulation of circadian rhythm. This study revealed the dynamic changes of histone modifications at the *Per* locus specifically in clock neurons. Importantly, it provides insights into the role of epigenetic factors in the regulation of dynamic gene expression changes in circadian rhythm.

## 1. Introduction

Studies in recent decades have revealed the core molecular mechanism that controls biological rhythms. The first molecular clock gene, *Period* (*Per*), was identified through a genetic screen of mutants generated in *Drosophila melanogaster* [1]. In *Drosophila* clock neurons, the protein complexes composed of CLOCK/CYCLE (CLK)/(CYC) and PERIOD/TIMELESS (PER)/(TIM) form a negative feedback loop [2]. Studying the regulatory mechanisms of the core clock genes within this regulatory loop is of great significance for improving our understanding of the molecular mechanisms of the biological clock.

Histone modification plays an important role in the regulation of circadian rhythm. H3K9me3 and H3K27me3 have been found to modify mammalian clock genes and their downstream genes [3–5]. Moreover, histone acetylation and ubiquitination are also important in circadian rhythm regulation [6–10]. However, most studies investigating the interplay between histone methylation/acetylation and clock regulation have been conducted in liver tissue or mammalian cell lines. The roles of histone methylation/acetylation in the core clock regulatory circuit of clock neurons are still unclear.

The analysis of the molecular features of clock neurons shows that they possess a unique pool of expressed genes. In a previous study, clock neuron-specific expressed genes were analysed using microarray technology [11]. However, the epigenetic characteristics of clock neurons remain unknown. Recently, the development of the mini-INTACT (isolation of nuclei tagged in specific cell type) method has provided a rapid way to isolate neurons for the chromatin immunoprecipitation (ChIP) experiment [12]. Moreover, the modified ChIP protocol made it possible to identify histone modifications using fewer than 1000 cells [13]. These protocols made it possible to access the chromosomal status of specific neuronal clusters. Identifying the histone modification status of clock neurons is important for understanding the role of the epigenetic regulation mechanism in the clock regulatory loop.

Evidence indicates that CoREST plays a role in the regulation of multiple histone modifications. Previous results show that CoREST can promote the demethylation of nucleosome substrate, while hyperacetylated nucleosomes are less susceptible to CoREST/LSD1-mediated demethylation [14,15]. Structural analysis demonstrates that CoREST possesses a flexible, bi-lobed structure with the two enzymes, LSD1 and HDAC1, located at opposite ends [16,17]. CoREST is reported to be involved in the CtBP-related complex, which can mediate H3K27 and H3K9 histone methylations [18]. Moreover, *in vivo* studies have shown that CoREST negatively regulates H3K27me3 in *Drosophila* follicle cells [19]. Interestingly, in the nervous system, activity-dependent changes in the short and long forms of CoREST within the CoREST/HDAC1 complex regulate memory flexibility in *Drosophila* [20]. However, it remains unclear whether CoREST contributes to the dynamic changes of histone modifications during circadian rhythm at different time points and the mechanisms underlying this regulation.

In this study, we found that *CoREST* plays a crucial role in the regulation of circadian rhythm. Through tissue-specific ChIP assays conducted in clock neurons, we found that *CoREST* regulates the levels of H3K27me3, H3K4me2, H3K27ac, H3K9me3 and H3K36me3 in these neurons. By identifying genetic and protein–protein interactions, we provided evidence that *CoREST* controls these histone modifications by regulating E(Z), HDAC1, KDM4A and LSD1. Notably, we also found that *CoREST* influences the binding of CLK to the *Per* locus. Finally, we proposed a working model that elucidates the role of the CoREST complex in regulating *Per* expression by integrating multiple histone modifications in clock neurons.

## 2. Material and methods

### 2.1. Fly stocks

The *CoREST*^*MI08173*^ mutant was generated through insertional mutagenesis (insert on X: 19530758) [21]. The following fly lines were obtained from the Bloomington Stock Center: *CoREST*^*MI08173*^ (no. 51221), *CoREST*^*EY14216*^ (no. 20793), *w*^*1118*^ (no. 5905), *HDAC1*^*303*^ (no. 26791), *KDM4A*^*KO*^ (no. 76241) and *UAS-CoREST F* (no. F000664). The following fly lines were obtained from the VDRC Stock Center: *UAS-CoREST F RNAi* (no. 34180), *UAS-HDAC1 RNAi* (no. 30599), *UAS-KDM4A RNAi* (no. 32650) and *UAS-LSD1 RNAi* (no. 25218). *E(Z)*^*63*^*/TM3* was kindly provided by Dr Alan Jian Zhu’s lab (Peking University, China). *Tim-Gal4* was a gift from Dr Yi Rao’s lab (Peking University, China). *UAS-CoREST RC RNAi* and *UAS-CoREST RC* were generated by Dr Hirano’s lab [20]. *3×UAS-unc84-GFP* was provided by Dr Agrawal [12]. *Clk-GFP; clk*^out^ was obtained from Dr Paul Hardin’s lab [22]. All fly lines used in this study were out crossed with the *w*^1118^ line to ensure a uniform genetic background.

### 2.2. *Drosophila* activity monitor-based method for circadian rhythm measurement

For all activity measurements, flies were kept in a 12 h light/12 h dark (12:12 LD) cycle at 25°C. Flies (3−5 days old) were individually loaded into detection tubes (length, 65 mm; inner diameter, 5 mm) containing standard cornmeal fly food at one end and a cotton stopper placed at the other end. The circadian rhythm was measured using the DAM (*Drosophila* activity monitor) System (Trikinetics, MA), which counted the infrared beam crossings of individual flies in each tube every 1 min. All circadian rhythm tests were carried out on male flies unless otherwise specified. Flies were entrained in the detection tube at 25°C for 72−96 h in a 12 h light/12 h dark cycle. Subsequently, data were collected in dark conditions for at least 5 days using the DAM System. The analyses of circadian rhythm were carried out using faasX software (obtained from the website https://trikinetics.com) and MATLAB (MathWorks, Natick, MA).

### 2.3. Circadian behaviour assessment of temperature-mediated phase shift

**Figure 1.**
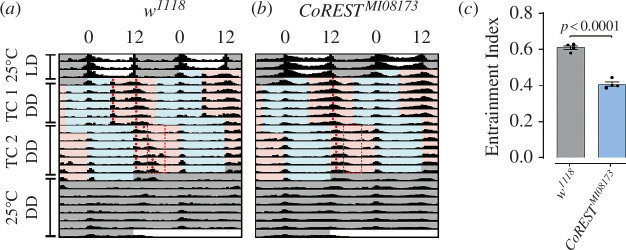
CoREST is required to synchronize with low temperature cycles in DD. (*a,b*) Actograms. Flies were initially synchronized to 3 LD cycles at 25°C, followed by two at 16°C and 25°C temperature cycles (TCs) in DD, each TC was delayed by 6 h compared to the previous regime, and subsequently released to DD at 25°C. Cyan and pink areas indicate 16 and 25°C, respectively. *n* = 16. Red dots indicate activity peaks. The red rectangle frames a 6 h window for the main temperature-synchronized activity. (*c*) Plotting of the EI. Data information: Statistical differences were measured using unpaired two-tailed Student’s *t*-test.

The temperature cycle (TC) experiment was conducted following the method described by Gentile *et al*. [23]. Initially, flies were synchronized to three light/dark (LD) cycles at 25°C. Subsequently, the temperature was reduced to 16°C for 6 h, followed by 12 h at 25°C and 12 h at 16°C TC for 6 days. The initial TC was then modified by delaying the temperature rise by 6 h, and flies were tested to resynchronize with the shifted TC for another 6 days. The entrainment index (EI) was calculated as the ratio of total activities occurring during a 6 h window to the activities occurring during the entire warm phase [23]. A value close to 1 indicates that most of the activity occurred within the specified window, indicating entrainment. Based on the inspection of the average activity profiles of the control flies, a 6 h window for the main temperature-synchronized activity was defined as ZT15–ZT20.5 (displayed as a red dotted line in figure 1*a*,*b*; ZT, zeitgeber time) [23].

### 2.4. Quantitative real-time PCR

Total RNA was extracted from 30 heads using TRIzol Reagent (TIANGEN, no. DP4−02). Reverse transcription and real-time PCR (RT-PCR) were performed using the PrimeScriptTM RT reagent Kit with gDNA Eraser (Perfect Real Time; TakaRa, no. RR047A) and SuperReal PreMix Plus (SYBR Green; TIANGEN, no. FP205−02) following the manufacturer’s instructions. All experiments were performed using the StepOne Real-Time PCR system (Applied Biosystem, Foster, CA). Quantification was performed using the ΔΔCT method. Unpaired two-tailed Student’s *t*-test (Prism GraphPad) was used to compare the differences between genotypes. All primers used are listed in electronic supplementary material, table S1. All quantitative RT-PCR analyses were performed with three biological replicates.

### 2.5. Clock neuron-specific chromatin immunoprecipitation

The genotypes used for the experiments were (i) *tim-Gal4/+; 3×UAS-unc84-GFP,* (ii) *CoREST^MI08173^/Y; tim-Gal4/+; 3×UAS-unc84-GFP,* (iii) *tim-Gal4/KDM4A^KO^; 3×UAS-unc84-GFP,* (iv) *tim-Gal4/+; 3×UAS-unc84-GFP/E(Z)^63^,* and (v) *tim-Gal4/+; 3×UAS-unc84-GFP/HDAC1^303^*. Newly enclosed flies were placed on standard fly food for 3–5 days, and then the entrained flies were collected at the indicated time point (ZT8 and ZT16, local time 14:30 and 22:30). Nuclei were obtained from 200 to 300 male heads using mini-INTACT protocol [12]. Rabbit anti-GFP (Invitrogen, no. G10362) was used in the mini-INTACT protocol. Then CNS ChIP for histone modification on *Per* genomic locus were performed using protocols described previously [13]. Anti-Histone H3 (tri methyl K9) (Abcam, no. ab8898), Anti-trimethyl Histone H3 (Lys27) (Millipore, no. 07–449), Anti-Histone H3 (acetyl K27) (Abcam, no. ab4729) and Tri-Methyl-Histone H3 (Lys36) (cell signalling technology, no. 4909s) were used in CNS ChIP. All CNS ChIP analyses were repeated three times with independent biological replicates. The percentage input was calculated by the following formula:


$$\%\;\text{input}=100^{*}2^{\wedge }\text{(−(Ct [ChIP] − (Ct [Input] -Log2 (Input 146 Dilution Factor)))}.$$

The primer pairs used for qPCR were listed in electronic supplementary material, table S1.

### 2.6. Cell culture and transient transfection

S2 cells were cultured in serum-free insect cell culture medium (HyClone, no. SH30278.02) at 25°C. Transient transfection was performed using X-tremeGENE HP DNA Transfection Reagent (Roche, no. 06366236001), following the manufacturer’s protocol.

### 2.7. Plasmid constructions

#### 2.7.1. Cloning of pAC-CN fusion plasmids

The N-terminal part of mCerulean (CN) was amplified via PCR from the pGWAAV-CMV-PSD95-mCerulean using the forward primer EcoRI-CN F and reverse primer NotI-CN R [24]. Plasmid pAC-mGFP was digested with EcoRI-NotI, and the CN fragment was inserted, resulting in pAC-CN. The *CoREST*-RF (*CoREST* long form) was amplified via PCR from the cDNA of fly heads using the forward primer NotI-CN-*CoREST*-RF F and the reverse primer HindIII-CN-*CoREST*-RF R. Plasmid pAC-CN was digested with NotI-HindIII, and the *CoREST*-RF fragment was inserted, resulting in pAC-CN-*CoREST*-RF. *LSD1*, *HDAC1* and *KDM4A* were also cloned into the pAC-CN vector using the same procedure, respectively. All primers used are listed in electronic supplementary material, table S1.

#### 2.7.2. Cloning of pAC-CC fusion plasmids

The C-terminal portion of mCerulean (CC) was amplified via PCR from the pGWAAV-CMV-PSD95-mCerulean using the forward primer NotI-CC F and reverse primer HindIII-CC R [24]. Plasmid pAC-mGFP was digested with NotI-HindIII, and the CC fragment was inserted, resulting in pAC-CC. The *CoREST*-RF was amplified via PCR from the cDNA of fly heads using the forward primer EcoRI-*CoREST*-RF-CC F and reverse primer NotI-*CoREST*-RF-CC R. Plasmid pAC-CC was digested with EcoRI-NotI, and the *CoREST*-RF fragment was inserted, resulting in pAC-*CoREST*-RF-CC. *E(Z*), *HDAC1* and *KDM4A* were also cloned into the pAC-CC vector using the same procedure, respectively. All primers used are listed in electronic supplementary material, table S1.

### 2.8. Bimolecular fluorescence complementation

The bimolecular fluorescence complementation (BiFC) analysis was performed following the method described by Bischof *et al*. [24]. Briefly, the mCerulean partial sequences encoding amino acid residues 1−173 (CN) or amino acid residues 173−238 (CC) were used to construct plasmids containing fusion genes. Fusion gene plasmids were transfected into S2 cells. After 48 h of transfection, cells were washed three times with phosphate-buffered saline (PBS). The samples were analysed using confocal microscopy (Leica SP8).

### 2.9. Chromatin immunoprecipitation to detect clock binding

Chromatin immunoprecipitation (ChIP) of adult heads was performed as previously described [25], with minor modifications. Twenty-five heads were collected in 450 μl PBS on ice. For cross-linking, 6.02 μl 37% formaldehyde was added, followed by incubation at room temperature for 10 min. Chromatin was sonicated for 2.5 min on ice (settings were 10 s on, 30 s off, high power). The sheared chromatin had an average length of 0.1–0.5 kb. Rabbit anti-GFP (Invitrogen, no. G10362) was used for immunoprecipitation. Fold enrichment was calculated by the ΔΔCT method. All ChIP analyses were repeated three times as independent biological replicates (refer to electronic supplementary material, table S1 for primer sequences).

### 2.10. Western blots

Three-day-old male fly heads were collected at indicated time points, and protein was extracted from approximately 30 heads using the RIPA buffer (150 mM NaCl, 1.0% NP-40, 0.5% sodium deoxycholate, 0.1% SDS, 50 mM Tris-HCl, pH 8.0). To prevent protein degradation, a protease inhibitor cocktail (CWBIO, no. CW2200) instructed by the manufacturer was also added to the buffer. The samples were then run on 8% SDS-polyacrylamide mini-gels and transferred onto 0.22 µm PVDF membranes (Millipore, no. ISEQ85R). Overnight incubation at 4°C was carried out with Anti-PER (Gift from Dr Jeffrey Price’s laboratory; 1:10000) and anti-β-Tubulin (ABclonal, no. AC008; 1:1000) on the membranes, respectively. HRP-labelled secondary antibodies (ABclonal, no. AS003) were diluted at 1:1000 and incubated for 4 h at room temperature. The signals were detected using ECL (ABclonal, no. RM00021P) by Amersham ImageQuant 800 (GEHealthcare, Sweden). The signal intensity was quantified by ImageJ software (NIH). The relative normalization of *Per* was normalized to the β-tubulin signal.

## 3. Results

### 3.1. *CoREST* long isoform was required for the regulation of circadian rhythm

**Figure 2.**
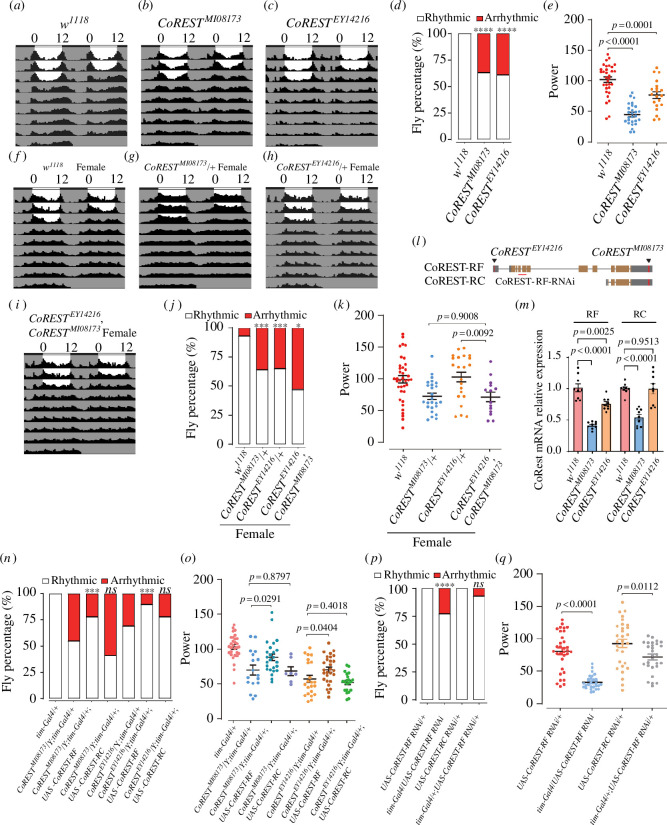
*CoREST* is involved in circadian rhythm regulation in adult *Drosophila*. (*a)–(c*) Representative locomotor activity over 5 days of *w^1118^*, *CoREST^MI08173^* and *CoREST^EY14216^* under constant darkness. (*w^1118^ n* = 16, *CoREST^MI08173^ n* = 16, *CoREST^EY14216^ n* = 16). (*d*) Percentage of rhythmic and arrhythmic flies in *w^1118^* (*n* = 32), *CoREST^MI08173^* (*n* = 43) and *CoREST^EY14216^* (*n* = 33). *p*-values of mutants compared to *w^1118^* using Fisher’s exact test are illustrated, where ****p < 0.0001. (*e*) Circadian power value shows the strength of circadian oscillation, individual flies with power (≥10) and ‘width’ value of 1.5 or more (representing the peak number within 30 min increments above the 95% confidence line of the periodogram) were considered rhythmic. Fly number is the same as (*d*). Statistical differences were measured using unpaired two-tailed Student’s *t*-test. (*f–i*) Representative locomotor activity over 5 days of *w^1118^* (female), *CoREST^MI08173^*/+ (female), *CoREST^EY14216^*/+ (female) and *CoREST^EY14216^*, *CoREST^MI08173^* (female) under constant darkness. (*w*^1118^ *n* = 16, *CoREST^MI08173^*/+ *n* = 16, *CoREST^EY14216^*/+*n* = 16, *CoREST^EY14216^*, *CoREST^MI08173^ n* = 16). (*j*) Percentage of rhythmic and arrhythmic flies in *w^1118^* (female, *n* = 40), *CoREST^MI08173^*/+ (female, *n* = 39), *CoREST^EY14216^*/+ (female, *n* = 34) and *CoREST^EY14216^*, *CoREST^MI08173^* (female, *n* = 30) under constant darkness. *p*-values of *CoREST^MI08173^*/+ and *CoREST^EY14216^*/+ compared with *w^1118^* using Fisher’s exact test are illustrated, where ***p < 0.001. *p*-values of *CoREST^EY14216^*, *CoREST^MI08173^* compared to *CoREST^MI08173^*/+ and *CoREST^EY14216^*/+ using Fisher’s exact test are illustrated, where *p < 0.05. (*k*) Circadian power value, fly number is the same as (*j*). Statistical differences were measured using unpaired two-tailed Student’s *t*-test. (*l*) CoREST has two main alternative splicing forms. (*m*) The expression of *CoREST RF* was reduced in *CoREST* mutants. Statistical differences were measured using unpaired two-tailed Student’s *t*-test. (*n*) Percentage of rhythmic and arrhythmic flies in *CoREST*^*MI08173*^*; tim-Gal4/+* (*n* = 31), *CoREST*^*MI08173*^*; tim-Gal4/UAS-CoREST RF* (*n* = 32), *CoREST*^*MI08173*^*; tim-Gal4/UAS-CoREST RC* (*n* = 30), *CoREST*^*EY14216*^*; tim-Gal4/+* (*n* = 32), *CoREST*^*EY14216*^*; tim-Gal4/UAS-CoREST RF* (*n* = 31), *CoREST*^*EY14216*^*; tim-Gal4/UAS-CoREST RC* (*n* = 27) under constant darkness. *p*-values of *CoREST*^*MI08173*^*; tim-Gal4/UAS-CoREST RF* and *CoREST*^*MI08173*^*; tim-Gal4/UAS-CoREST RC* compared with *CoREST*^*MI08173*^*; tim-Gal4/+* using Fisher’s exact test are illustrated, where *ns* indicates no significant difference, ***p < 0.001. *p-*values of *CoREST*^*EY14216*^*; tim-Gal4/UAS-CoREST RF* and *CoREST*^*EY14216*^*; tim-Gal4/UAS-CoREST RC* compared with *CoREST*^*EY14216*^*; tim-Gal4/+* usingFisher’s exact test are illustrated, where *ns* indicates no significant difference, ***p < 0.001. (*o*) Circadian power value, fly number is the same as (*n*). Statistical differences were measured using unpaired two-tailed Student’s *t*-test. (*p*) Percentage of rhythmic and arrhythmic flies in *UAS-CoREST RF RNAi/+* (*n* = 32), *tim-Gal4/UAS-CoREST RF RNAi/+* (*n* = 35), *UAS-CoREST RC RNAi/+* (*n* = 30) and *tim-Gal4/+; UAS-CoREST RC RNAi/+* (*n* = 30) under constant darkness. *p*-values of *tim-Gal4/+; UAS-CoREST RF RNAi/+* compared with *UAS-CoREST RF RNAi/+* usingFisher’s exact test are illustrated, where ****p < 0.0001. *p*-values of *tim-Gal4/+; UAS-CoREST RC RNAi/+* compared with *UAS-CoREST RC RNAi/+* usingFisher’s exact test are illustrated, where *ns* indicates no significant difference. (*q*) Circadian power value, fly number is the same as (*p*). Statistical differences were measured using unpaired two-tailed Student’s *t*-test.

In a candidate screen aimed at identifying epigenetic regulators of circadian rhythm, we discovered that mutations of *CoREST* resulted in circadian rhythm defects. The two insertional mutations of *CoREST*, *CoREST^MI08173^* and *CoREST^EY14216^*, resulted in a decrease in rhythmicity percentages to 62.8% and 60.6% respectively, compared with *w*^*1118*^ control (figure 2*a*,*d*). Moreover, both mutants exhibited significantly reduced power values (figure 2*e*). Female flies carrying *CoREST^MI08173^* and *CoREST^EY14216^* mutations also exhibited identical circadian rhythm defects as observed in male flies (figure 2*f*,*h*,*j*). Furthermore, the weak enhancement of circadian phenotypes in the *CoREST^MI08173^*/*CoREST^EY14216^* double mutant (female; both genes are on the X chromosome) (figure 2*i*,*k*) suggested that these two alleles likely share similar mechanisms in causing circadian phenotypes. Hence, these results support the role of *CoREST* as a regulator of circadian rhythm.

A previous study has shown that *CoREST* has two major splicing forms with different functions, which are represented by RC and RF (figure 2*l*) [20]. the long form of *CoREST* was referred to as *CoREST-RF*. While, the N-terminus truncated isoform was *CoREST-RC*, which contains an extra sequence at the 5′UTR (figure 2*l*). *CoREST^EY14216^* and *CoREST^MI08173^* alleles are insertional mutations of *CoREST*, inserted in the 5′ and 3′ of the *CoREST* coding region, respectively (figure 2*l*). As a result, *CoREST^EY14216^* only affects RF instead of RC (figure 2*m*). On the other hand, *CoREST^MI08173^* affects both the RF and the RC isoforms (figure 2*m*).

To determine which splicing form was crucial in the circadian phenotypes caused by *CoREST^MI08173^* and *CoREST^EY14216^* alleles, we conducted genetic interaction experiments. Our findings revealed that the expression of RF driven by *tim*-Gal4 was able to rescue the circadian phenotypes caused by both alleles (figure 2*n*,*o*). In contrast, the expression of RC driven by *tim*-Gal4 failed to rescue the phenotypes caused by *CoREST^MI08173^* and *CoREST ^EY14216^* (figure 2*n*,*o*). Consistently, specific RNAi knockdown of RC did not result in noticeable circadian phenotypes (figure 2*p*,*q*). The knockdown of *CoREST* RF in clock neurons using *tim*-Gal4 resulted in a decrease in the percentage of rhythmicity to 77.1% (figure 2*p*). Moreover, the power value was also significantly reduced in *tim*-Gal4/UAS-*CoREST RF RNAi* (figure 2*q*). These results indicated that the long-form RF was the major isoform responsible for the circadian phenotypes.

In conclusion, *CoREST* long isoform was essential for regulating the circadian rhythm. In our subsequent experiments, we primarily used the *CoREST^MI08173^* allele as it exhibits stronger effects compared to the other allele.

### 3.2. *CoREST* regulates circadian rhythm by modulating the expression of *Per*

Previous studies have reported that *CoREST* mediates the binding of epigenetic factors on chromatin [16,17,26]. To investigate the mechanism of *CoREST* function in circadian rhythm, we examined the binding profile of epigenetic factors on core clock regulators, *Per*, *Clk*, *Tim* and *Cyc*. In modENCODE database (http://www.modencode.org/), we identified significant binding of KDM4A, LSD1 and HDAC1 on the *Per* locus (electronic supplementary material, figure S1A) [27]. Previous data have shown that LSD1 and HDAC1 are binding proteins of CoREST [16,17,26]. It has also been observed that LSD1 interacts with E(Z) [28,29]. Moreover, previous studies have shown that mutation and overexpression of *Per* result in strong and weak rhythmic phenotypes, respectively [1,30]. Therefore, we hypothesized that CoREST regulates circadian rhythm by controlling the activity of these factors at the *Per* locus.

**Figure 3.**
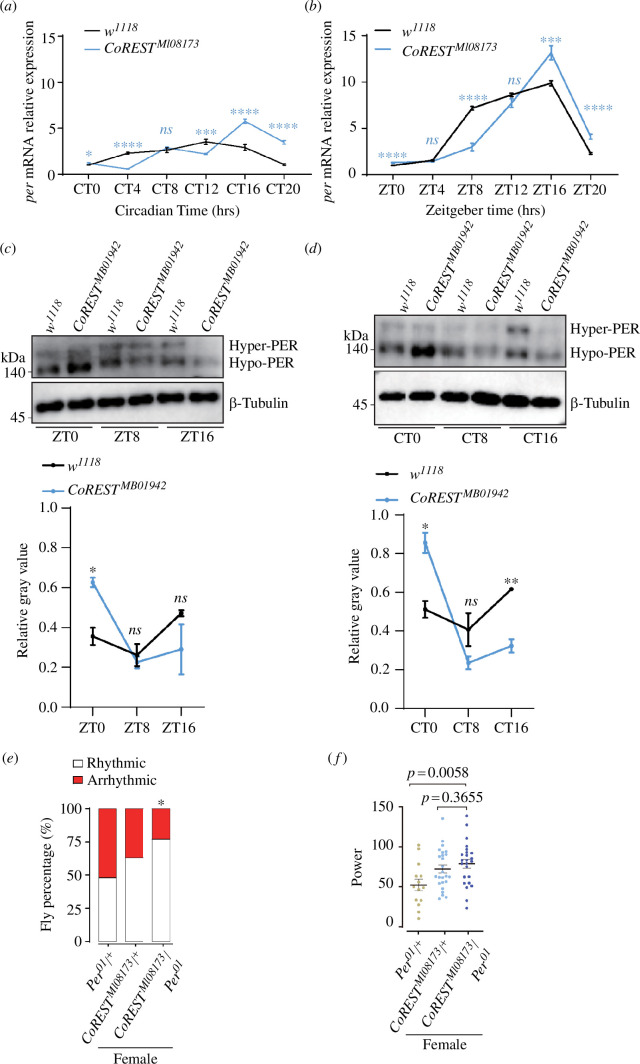
*CoREST* modulated *Per* expression. (*a,b*) Quantitative RT-PCR of *Per* in adult heads of *CoREST^MI08173^* and *w^1118^* under DD and LD, respectively. Single time point data comparison analysis was performed by unpaired two-tailed Student’s *t*-test, *ns* indicates no significant difference, *p < 0.05, ***p < 0.001 and ****p < 0.0001. We also used JTK_CYC to analyse the expression oscillations of *Per. w^1118^* JTK_amplitude = 0.449, *p* < 0.0001; *CoREST^MI08173^* JTK_amplitude = 1.500, *p* = 0.0008 for (*a*). *w^1118^* JTK_amplitude = 4.631, *p* < 0.0001; *CoREST^MI08173^* JTK_amplitude = 5.460, *p* = 0.0001 for (*b*). (*c,d*) The mutation of *CoREST* altered the oscillation of PER protein. The antibody against β-tubulin served as the loading control. The relative grey value of western blot was measured by ImageJ. Statistical differences were measured using unpaired *t-*test. *ns* indicates no significant difference, *p < 0.05 and **p < 0.01. (*e*) Percentage of rhythmic and arrhythmic flies in *per^01^*/+female (*n* = 29), *CoREST^MI08173^*/+ female (*n* = 39) and *per^01^*/*CoREST^MI08173^* female (*n* = 31) under constant darkness. *p*-values of *per^01^*/*CoREST^MI08173^* female compared with *CoREST^MI08173^*/+ female using Fisher’s exact test are illustrated, where *p < 0.05. (*f*) Circadian power value, fly number is the same as (*e*). Statistical differences were measured using unpaired two-tailed Student’s *t*-test.

To test our hypothesis, we examined the effects of *CoREST* mutation on the expression of *Per*. First, we detected the relative expression level of *Per* in *CoREST^MI08173^*. We found that under constant darkness (CT) conditions, the peak of *Per* expression in *w*^*1118*^ was located at CT12, while this peak was shifted to CT16 in *CoREST^MI08173^* (figure 3*a*). Compared with the *w^1118^*, the overall oscillation pattern of *Per* expression in *CoREST^MI08173^* was significantly enlarged (figure 3*a*; *w*^*1118*^ JTK_amplitude = 0.449, *p* < 0.0001; *CoREST^MI08173^* JTK_amplitude = 1.500, *p* = 0.0008) [31]. Under the LD condition, the peaks of *Per* expression of *w^1118^* and *CoREST^MI08173^* were at ZT16 (figure 3*b*). Compared with the *w^1118^*, the overall oscillation pattern of *Per* expression in *CoREST^MI08173^* was also significantly enlarged (figure 3*b*; *w*^*1118*^ JTK_amplitude = 4.631, *p* < 0.0001; *CoREST^MI08173^* JTK_amplitude = 5.460, *p* = 0.0001). Consistently, the variation of *Per* expression level from ZT8 to ZT16 was steeper compared with the *w^1118^* (figure 3*b*). The protein level of Per was also influenced by *CoREST*. The detection of PER protein at various time points demonstrated alterations in its protein level (figure 3*c*,*d*). These results imply that *CoREST* influences the expression pattern of *Per*. In addition, we also constructed *CoREST^MI08173^/Per^01^* double mutant (female) to reduce the amplitude of *Per* and performed behavioural assays. The results showed that the *CoREST^MI08173^/Per^01^* double mutant could partially rescue the circadian phenotype caused by *CoREST^MI08173^* (figure 3*e*,*f*). Based on these results, we concluded that *CoREST* regulates circadian rhythm by modulating *Per* expression.

### 3.3. Identification of genetic and physical interactions between *CoREST* and histone modification factors KDM4A, LSD1, E(Z) and HDAC1

To further validate the hypothesis that CoREST regulates the circadian rhythm by recruiting epigenetic factors (including KDM4A, LSD1, E(Z) and HDAC1) at the *Per* locus, we conducted genetic interactions tests between CoREST and these histone modification factors. We examined the circadian rhythm phenotypes of various mutants and double mutants. The results showed that the mutations in *KDM4A* and *HDAC1* resulted in mild rhythmic defects, respectively (with the percentage of rhythmicity at 88.5% in *KDM4^KO^*/+ and 96.6% in *HDAC1^303^/+*) while, the mutation in *E(Z*) did not result in any rhythm defects (percentage rhythmicity in *E(Z)^63^/+* was 100%) (electronic supplementary material, figure S1*b, f*). We deduced that the weaker rhythm phenotype occurred because none of these mutants could be homozygous, and only a subset of them could fulfil the organism’s required function. Consistently, the knockdown of *LSD1*, *KDM4A* and *HDAC1* in clock neurons resulted in mild defects in the percentage of rhythmicity (electronic supplementary material, figure S1*e*). In contrast, the power value significantly decreased, thereby confirming our conjecture (electronic supplementary material, figure S1*f*). These results suggest that *HDAC1*, *KDM4A* and *LSD1* are all involved in the regulation of circadian rhythm.

**Figure 4.**
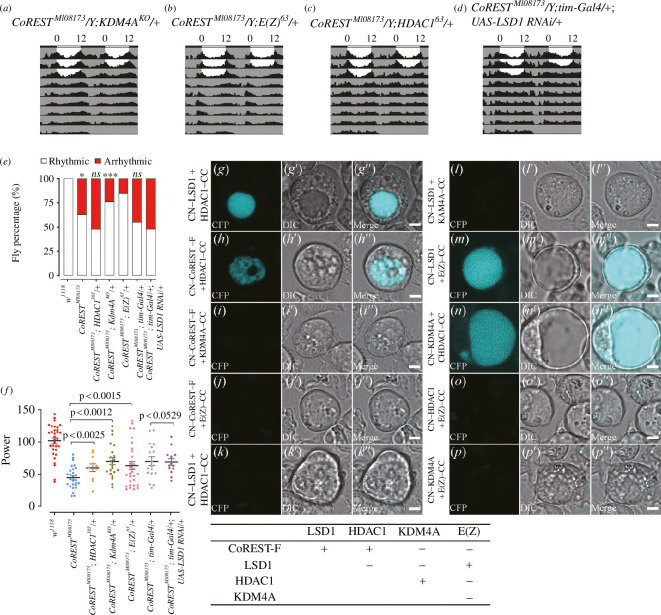
Genetic interactions of CoREST with various histone modification factors. (*a)–(d*) Representative locomotor activity over 5 days of *CoREST*^*MI08173*^*; KDM4A*^*KO*^*/+* (*n* = 16), *CoREST*^*MI08173*^*; E(Z)*^*63*^*/+* (*n* = 16), *CoREST*^*MI08173*^*; HDAC1*^*303*^*/+* (*n* = 16) and *CoREST*^*MI08173*^*; tim-Gal4/+; UAS-LSD1 RNAi/+* (*n* = 16) under constant darkness. (*e*) Percentage of rhythmic and arrhythmic flies in *w^1118^* (*n* = 32), *CoREST^MI08173^* (*n* = 43), *CoREST*^*MI08173*^*; HDAC1*^*303*^*/+* (*n* = 23), *CoREST*^*MI08173*^*; KDM4A*^*KO*^*/+* (*n* = 29), *CoREST*^*MI08173*^*; E(Z)*^*63*^*/+* (*n* = 34) and *CoREST*^*MI08173*^*; tim-Gal4/+; UAS-LSD1 RNAi/+* (*n* = 31) under constant darkness. *p-*values of *CoREST*^*MI08173*^*; HDAC1*^*303*^*/+*, *CoREST*^*MI08173*^*; KDM4A*^*KO*^*/+* and *CoREST*^*MI08173*^*; E(Z)*^*63*^*/+* compared with *CoREST^MI08173^* using Fisher’s exact test are illustrated, where *ns* indicates no significant difference, *p < 0.001, ***p < 0.001. *p*-values of *CoREST*^*MI08173*^*; tim-Gal4/+; UAS-LSD1 RNAi/+* compared with *CoREST*^*MI08173*^*; tim-Gal4/+* using Fisher’s exact test are illustrated, where *ns* indicates no significant difference. (*f*) Circadian power value, fly number is the same as (*e*). Statistical differences were measured using unpaired two-tailed Student’s *t*-test. (*g–p'*)') Two vectors carrying different genes were co-transfected into S2 cells. (*g–g''*) CN-LSD1 and CoREST-F-CC were co-transfected into S2 cells. (*h–h''*) CN-CoREST-F and HDAC1-CC were co-transfected into S2 cells. (*i–i''*) CN-CoREST-F and KDM4A-CC were co- transfected into S2 cells. (*j–j''*) CN-CoREST-F and E(Z)-CC were co-transfected into S2 cells. (*k–k''*) CN-LSD1 and HDAC1-CC were co-transfected into S2 cells. (*l–l''*) CN-LSD1 and KDM4A-CC were co-transfected into S2 cells. (*m–m''*) CN-LSD1 and E(Z)-CC were co-transfected into S2 cells. (*n–n''*) CN-KDM4A and HDAC1-CC were co-transfected into S2 cells. (*o–o''*) CN-HDAC1 and E(Z)-CC were co-transfected into S2 cells. (*p–p''*) CN-KDM4A and E(Z)-CC were co-transfected into S2 cells. Scale bar: 3 µm. (*q*) Summary of the interactions between the proteins that make up the CoREST complex. ‘+’ indicates physical interaction between two proteins. ‘−’ indicates that there is no physical interaction between two proteins.

*CoREST^MI08173^; KDM4A^KO^*/+double mutant (percentage of rhythmicity was 75.9% in the double mutant compared to 62.8% in *CoREST^MI08173^*) did not significantly rescue or enhance the percentage of rhythmicity of *CoREST^MI08173^* (figure 4*a*,*e*). However, *CoREST^MI08173^; E(Z)^63^*/+double mutant (percentage of rhythmicity was 85.3% in the double mutant compared to 62.8% in *CoREST^MI08173^*) showed the rescued phenotypes of *CoREST^MI08173^* in the percentage of rhythmicity (figure 4*b*,*e*). There was also a clear enhancement in the power value (figure 4*f*).

*CoREST^MI08173^; HDAC1^303^* double mutant (percentage of rhythmicity was 47.8% in the double mutant compared to 62.8% in *CoREST^MI08173^*) showed an enhanced phenotype of *CoREST^MI08173^* in the percentage of rhythmicity (figure 4*c*,*e*). *CoREST^MI08173^; tim*-Gal4/+; UAS-*LSD1 RNAi/+* (percentage rhythmicity of 48.4% compared with 54.8% in *CoREST^MI08173^; tim-*Gal4/+) showed a trend towards an enhanced phenotype of *CoREST^MI08173^* in the percentage of rhythmicity and power value (figure 4*d*,*f*). These results indicate that these factors are potential circadian regulators that mediate the function of CoREST in clock neurons.

Physical interactions were found among CoREST, KDM4A, LSD1, E(Z) and HDAC1. To investigate the mechanism of *CoREST*’s impact on these histone-modifying factors, we conducted the BiFC assay to test their interaction properties (figure 4*g*,*p”*; electronic supplementary material, S1). The BiFC protein–protein interaction assay revealed that the long form of CoREST (CoREST-RF) interacted with both LSD1 an HDAC1, but not with KDM4A or E(Z) (figure 4*g–j” ,q”*; electronic supplementary material, S2*a*, *h*"). LSD1 was found to interact with E(Z), but not with HDAC1 or KDM4A (figure 4*k–m”*, *q”*; electronic supplementary material, S2*a*, *h″*). HDAC1 showed an interaction with KDM4A (figure 4*n–n",q*; electronic supplementary material, S2*a*, *h*″). However, E(Z) did not exhibit any interactions with HDAC1 or KDM4A (figure 4*o–q*; electronic supplementary material S2*a, h*″). Previous studies showed that CoREST-RF interacts with LSD1 and HDAC1 to facilitate their functions [14,15]. These results suggested the possibility of a complex formation involving CoREST-RF, HDAC1, LSD1, KDM4A and E(Z) through direct interactions between CoREST-RF and HDAC1, CoREST-RF and LSD1, LSD1 and E(Z), HDAC1and KDM4A.

### 3.4. In clock neurons, multiple histone modifications at the *Per* locus were affected by CoREST

**Figure 5.**
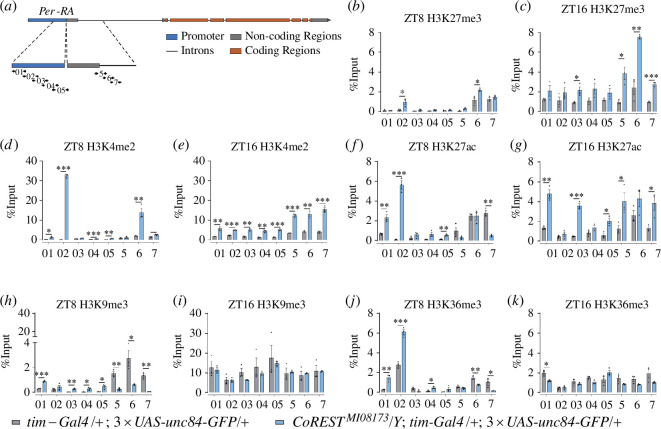
CoREST regulating histone modification on the *Per* genomic locus in clock neurons. (*a*) Primers used in (*b*)–(*k*). (*b,c*) ChIP experiments showing the relative enrichment (% Input) of H3K27me3 on the *Per* gene locus in *CoREST^MI08173^* and *w^1118^*. (*b*) H3K27me3 was weakly upregulated in *CoREST^MI08173^* at ZT8. (*c*) H3K27me3 was strongly upregulated in the *CoREST^MI08173^* mutant at ZT16. (*d,e*) ChIP experiments showing the relative enrichment (% Input) of H3K4me2 on *Per* gene locus in *CoREST^MI08173^* and *w^1118^*. (*d*) H3K4me2 was strongly upregulated in *CoREST^MI08173^* at ZT8. (*e*) H3K4me2 was also strongly upregulated in the *CoREST^MI08173^* mutant at ZT16. (*f,g*) ChIP experiments showing the relative enrichment (% Input) of H3K27ac on *Per* gene locus in *CoREST^MI08173^* and *w^1118^*. (*f*) H3K27ac was upregulated in the *CoREST^MI08173^* mutant at ZT8. (*g*) H3K27ac was also upregulated in the *CoREST^MI08173^* mutant at ZT16. (*h,i*) ChIP experiments showing the relative enrichment (% Input) of H3K9me3 on the *Per* gene locus in *CoREST^MI08173^* and *w^1118^*. (*h*) H3K9me3 was downregulated in the *CoREST^MI08173^* mutant at ZT8. (*i*) H3K9me3 was invariant in the *CoREST^MI08173^* mutant at ZT16. (*j,k*) ChIP experiments showing the relative enrichment (% Input) of H3K36me3 on the *Per* gene locus in *CoREST* mutant *CoREST^MI08173^* and *w^1118^* control. (*j*) H3K36me3 was upregulated in *Per* promoter but downregulated in *Per* gene body in the *CoREST^MI08173^* mutant at ZT8. (*k*) H3K36me3 was weakly downregulated in the *CoREST^MI08173^* mutant at ZT16. Data information: Statistical differences were measured using unpaired two-tailed Student’s *t*-test. The significance levels were represented as *p < 0.05, **p < 0.01, ***p < 0.001 and ****p < 0.0001. If the *p*-value was greater than 0.05, it was not displayed in the figures.

We deduce that if CoREST recruits these epigenetic factors at *Per* locus to regulate the circadian rhythm, the relevant histone modifications should rely on CoREST. Therefore, we conducted ChIP experiments in the context of clock neurons to investigate whether the histone modifications at the *Per* locus were altered owing to *CoREST* mutation. We analysed H3K27me3, regulated by E(Z) [32,33], H3K4me2 regulated by LSD1 [34], H3K27ac regulated by HDAC1 [35], H3K9me3 regulated by KDM4A and LSD1 [34,36] and H3K36me3 regulated by KDM4A [36,37] in the clock neurons of *w^1118^* and *CoREST^MI08173^* using CNS-ChIP (see §2) (figure 5*a*).

We found that at the *Per* locus, the negative control IgG showed no change. In contrast, H3K27me3 significantly increased at ZT16 compared with ZT8 in the control group (electronic supplementary material, figure S3*b, d*). After the *CoREST* mutation, there was a significant increase in the level of H3K27me3 (figure 5*b*,*c*), indicating that CoREST negatively regulates this modification. E(Z) is a positive regulator of H3K27me3 at both ZT8 and ZT16 (electronic supplementary material, figure S3c , *d*). These results suggest that the CoREST negatively regulates E(Z) in clock neurons, which is consistent with the results of the genetic interaction between *CoREST* and *E(Z*) mutants (figure 4*b,e, f*).

H3K4me2 levels were found to be higher at ZT16 than at ZT8 in the *Per* locus in the control group (figure 5*d*,*e*). The level of H3K4me2 was significantly higher in the *CoREST^MI08173^* mutant (figure 5*d*,*e*), indicating a negative regulation by CoREST. The core of the CoREST complex contains LSD1, a histone modification enzyme that demethylates histone H3K4-me2 and -me1 residues [14–17]. Therefore, these results suggest that the CoREST positively regulates LSD1 in clock neurons.

The levels of H3K27ac showed little change at the *Per* locus between ZT16 and ZT8 in control (electronic supplementary material, figure S3E and F). After *CoREST* mutation, the level of H3K27ac significantly increased (figure 5*f*,*g*), indicating a negative regulation by CoREST. HDAC1 was the negative regulator of H3K27ac at both ZT8 and ZT16 (electronic supplementary material, figure S3*e* , *f*). Consequently, CoREST positively regulates HDAC1, which is consistent with the results of genetic interaction between *CoREST* and *HDAC1* mutants (figure 4*c,e,f*).

At the *Per* locus, H3K9me3 levels were significantly higher at ZT16 compared with ZT8 in the control group (electronic supplementary material, figure S3G and H). After the *CoREST* mutation, H3K9me3 levels decreased significantly at ZT8, indicating that CoREST positively regulates H3K9me3 (figure 5*h*). However, there was no effect on H3K9me3 levels at ZT16 when the CoREST function was lost (figure 5*i*), indicating that the CoREST regulation of H3K9me3 was time dependent. To further explore whether the temporal specificity was owing to the time-dependent function of KDM4A, we examined H3K9me3 in KDM4A mutants. The results showed that KDM4A primarily acts as a negative regulator of H3K9me3 at ZT8, while it had no effect on H3K9me3 at ZT16 (electronic supplementary material, figure S3G and H). In addition, it has been reported that LSD1 facilitates H3K9me3 modification [34]. These results suggest that CoREST negatively regulates KDM4A, but positively regulates LSD1 in clock neurons, which is consistent with the results of genetic interaction analysis between *CoREST*, *KDM4A* and *LSD1* mutants (figure 4*e*,*f*).

H3K36me3 levels are higher at ZT16 than at ZT8 in the *Per* promoter in the control group (electronic supplementary material, figure S3I and J). After the *CoREST* mutation, there was a significant increase in H3K36me3 levels at ZT8 and a significant decrease at ZT16 (figure 5*j*,*k*). For H3K36me3, KDM4A acted as a positive regulator at ZT8 but a negative regulator at ZT16 (electronic supplementary material, figure S3*i*, *j*). Consequently, at both ZT8 and ZT16, CoREST negatively regulates KDM4A, which is consistent with the situation observed with H3K9me3. Similar to H3K9me3, the temporal-specific function of CoREST may be attributable to the time-dependent role of KDM4A. This conjecture is supported by the rescue of the *CoREST^MI08173^* power value by the *CoREST^MI08173^; KDM4A^KO^*/+double mutant (figure 4*a,e,f*).

To investigate whether *CoREST* regulates the cycling of histone modifications at the *period* locus in the clock neurons, we examined the H3K27me3 levels in clock neurons of both *CoREST* mutants and the controls. Consistent with the mRNA levels, we observed oscillations with a much higher amplitude in the mutants, reaching a peak at ZT16 (electronic supplementary material, figure S3*k*).

In conclusion, these results demonstrate that *CoREST* regulates circadian rhythm. This regulation was to some extent dependent on histone-modifying factors, such as KDM4A, LSD1, E(Z) and HDAC1, along with their corresponding histone modifications. Moreover, the temporal-specific regulation of H3K9me3 and H3K36me3 by CoREST can be attributed to the temporal-specific function of KDM4A.

### 3.5. CoREST complex regulates CLK binding at the *Per* locus

**Figure 6.**
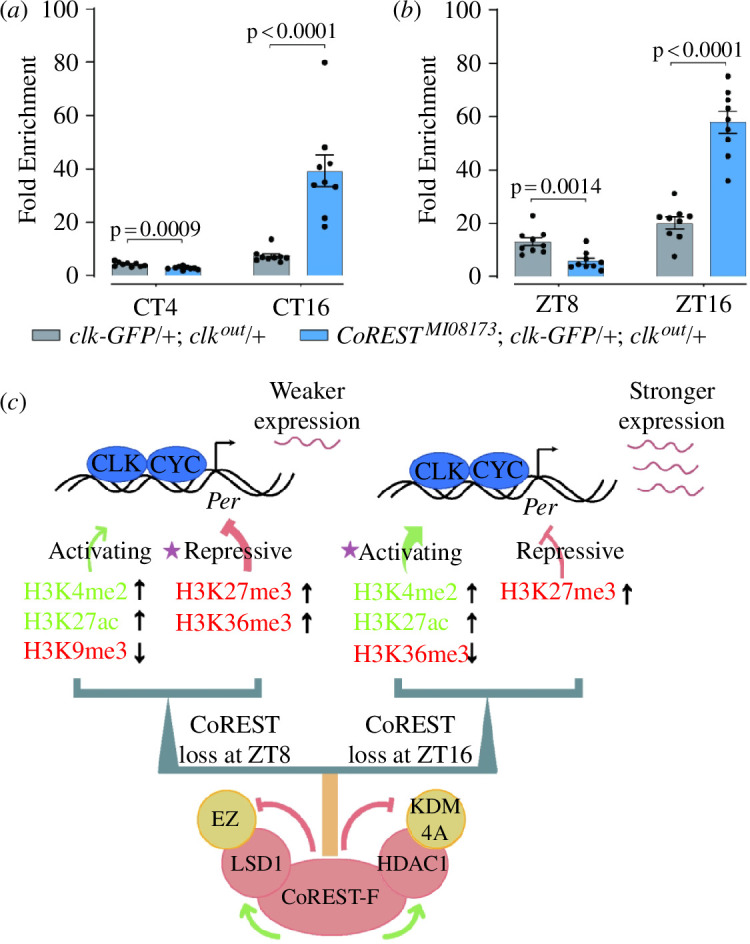
Dynamics of clock binding to *Per* E-boxes. (*a–b*) ChIP experiment was conducted to identify the binding of CLK on *Per* E-boxes. (*a*) Fold enrichment of CLK at CT4 and CT16. (*b*) Fold enrichment of CLK at ZT8 and ZT16. (*c*) Model depicting CoREST function. Data information: Statistical differences in (*a) and (b*) were measured using unpaired two-tailed Student’s *t*-test.

A previous study has shown that CLK is the main transcriptional factor of *Per* [38]. Epigenetic modifications often regulate gene expression through transcription factors. Therefore, we investigated whether CoREST regulates the binding of CLK to the *Per* gene locus. The expression level of *Per* at CT16 or ZT16 was significantly higher than that at CT4 or ZT8 (figure 3*a*,*b*). Consistent with this, the CLK binding at the *Per* locus of CT16 or ZT16 was more abundant compared to that at CT4 or ZT8 (figure 6*a*,*b*). In *CoREST* mutants, compared to the control group, the CLK binding at the *Per* locus of CT16 or ZT16 increased, while it decreased at CT4 or ZT8 (figure 6*a*,*b*), indicating that the presence of the CoREST complex inhibited CLK binding at CT16 and ZT16 while enhancing CLK binding at CT4 and ZT8. Therefore, the overall effect of CoREST complex regulation on the *Per* locus is to maintain the variation of CLK binding and the oscillation of *Per* expression within a narrow range.

In conclusion, we have a model illustrating the regulation of the CoREST complex on histone modification at the *Per* locus (figure 6*c*). Through direct interactions between CoREST-RF and HDAC1, CoREST-RF and LSD1, LSD1 and E(Z), HDAC1 and KDM4A, CoREST positively regulates LSD1 and HDAC1, while negatively regulates E(Z) and KDMA. Under the influence of these histone-modifying factors, CoREST interactively regulates multiple histone modifications in a time-dependent manner. At ZT8, the absence of CoREST led to a substantial elevation in H3K4me2 and H3K27ac, a significant decrease in H3K9me3, and an increase in H3K27me3 and H3K36me3, ultimately resulting in a decrease in *Per* expression (figures 3*b* and 6*c*). While, at ZT16, the loss of CoREST resulted in a significant elevation in H3K27me3, a mild decrease in H3K36me3, a significant increase in H3K4me2 and H3K27ac, and no significant change in the repressive mark H3K9me3. These combined effects contributed to an increase in *Per* expression (figures 3*b* and 6*c*). Notably, at ZT16, both the gene body and promoter of *Per* exhibited a substantial repressive modification, H3K9me3, which likely accounted for the subsequent trough in *Per* expression (figures 3b,5i and 6*c*).

### 3.6. The ability of *Drosophila* to be entrained by environmental changes is limited by *CoREST* mutation

To further investigate the function of *CoREST*-dependent epigenetic regulatory machinery in *Drosophila*, we examined the adaptation of the circadian rhythm of the *CoREST* mutant and the control group to changing conditions. Specifically, we tested the temperature entrainment condition at 16°C for 12 h followed by 12 h at 25°C TCs. The results revealed a significant reduction in the entrainment index for the *CoREST* mutations (figure 1*a*,*c*), indicating a decreased ability to adapt to environmental changes. It has been previously reported that synchronization to low TCs in DD requires Per in ventral lateral neurons [23]. The altered expression pattern of *Per* caused by *CoREST* mutations may explain the inability of CoRESTMI*^08173^* to adapt to environmental changes. In conclusion, these data collectively indicate that *CoREST* mutation limits the ability of *Drosophila* to be entrained by temperature.

## 4. Discussion

In this study, we investigated the mechanism of *CoREST* regulation in circadian rhythm. By studying the regulation of the CoREST complex on circadian rhythm, we revealed that CoREST regulates HDAC1, LSD1, E(Z) and KDM4A and their corresponding histone modifications at the *Per* locus specifically in clock neurons. Interestingly, we found dynamic changes in histone modifications at different time points of the *Per* locus, suggesting that histone modification plays an important role in regulating gene expression oscillation. More importantly, we found differential effects of the same factor on the *Per* locus at different time points, such as the effect of KDM4A on H3K9me3 and H3K36me3 at ZT8 and ZT16. Understanding the mechanism behind this time dependence poses an intriguing problem.

The regulatory relationships identified in this study are consistent with or provide an explanation for phenotypes previously reported in the literature. The discovery of CoREST’s function on E(Z) explains the previous report that CoREST negatively regulates H3K27me3 in *Drosophila* follicle cells [19]. The discovery of CoREST’s role on LSD1 and E(Z) also explains its involvement in regulating the H3K4me2, H3K9me3 and H3K27me3, as reported in a previous study [18]. Previous reports have indicated that H3K36me3 levels increase alongside transcription to inhibit repeated transcription [39]. Specifically, H3K36me3 on the gene body correlates with transcriptional activity. Conversely, the presence of H3K36me3, along with H3K9me3, at the regulatory region maintains a repressive state of gene expression [40]. Our study demonstrates a positive correlation between H3K36me3 on the gene body of the *Per* locus and transcriptional levels, while a negative correlation is observed between H3K36me3 at the regulatory region and transcriptional levels (figure 5*j*,*k*). All of these pieces of evidence support the effectiveness of the techniques employed in this study.

Histone modifications, as a feedback regulation mechanism, maintain the dynamic expression of *Per* in a relatively narrow range. Transcription is mainly driven by CLK. This study shows that the level of inhibitory histone modification is closely related to the *Per* transcriptional level in *w*^1118^ flies. There is a time delay between the mRNA level and the levels of epigenetic modifications. This phenomenon is similar to what we observe for the mRNA level and protein level of clock genes. At ZT8, when the expression level is relatively low, the inhibitory histone modifications are also low. This low level of inhibitory histone modifications at the *Per* locus facilitates the subsequent enhancement of the transcriptional level. On the contrary, at ZT16, when the expression level is relatively high, the inhibitory histone modifications are high. This high level of inhibitory histone modifications at the *Per* locus dampens its subsequent transcription. The function of CoREST complex is to maintain histone modifications of *Per* locus. Our data indicate that except for H3K9me3 at ZT16, the other four modifications can be altered by *CoREST* mutation. Other mechanisms are involved in maintaining H3K9me3 at ZT16. The collective effects of multiple histone modifications at the *Per* locus can influence the recruitment of CLK and transcription. The increase in activating histone modifications promote CLK binding and transcription, and vice versa (figure 6*c*). The question then remains: how is the initial state established with both a relatively high level of H3K9me3 and a relatively high CLK binding and transcription level? Feedback mechanisms are probably involved in this process.

One interesting discovery in this study is the temporal-specific role of CoREST in regulating H3K9me3 and H3K36me3. Further evidence from our study demonstrates that this is attributed to the temporal-dependent function of KDM4A. Regarding H3K9me3, the lack of impact from KDM4A or CoREST loss at ZT16 may be owing to high levels of H3K9me3 at this stage or the involvement of other mechanisms maintaining its level. Additionally, other similar mechanisms have been observed to maintain H3K9me3 levels in mammals [3]. As for H3K36me3, the contrary effects at ZT8 and ZT16 resulting from KDM4A or CoREST loss could be a consequence of different histone modification states, diverse expression patterns of interacting factors, or varying responses from redundant homologues during these time points. Investigating the underlying mechanism behind this phenomenon would be of great interest, although we are currently lacking the necessary reagents to conduct further experiments. This study serves as yet another example highlighting the involvement of the CoREST complex in timely dynamic transcriptional regulation. A previous report by Y.H.’s lab demonstrated the role of CoREST in activity-dependent transcription regulation in memory [20].

The tests of physical interactions among the factors mentioned in this study have limitations. The lack of an *in vivo* context means that the results shown here only provide evidence of the possibility of interaction, rather than the actual states of the protein complexes. As previously mentioned, the CoREST complex may exhibit dynamic behaviour at different time points in terms of temporal-specific regulation. The model in figure 6*c* may only capture a limited state of the CoREST complex. The inclusion of certain components, particularly E(Z) or KDM4A, could be subject to dynamic regulation by other factors. The genetic relationship discovered in this study is limited in this regard. It would be intriguing to further investigate the components and functions of the CoREST complex at various time points in clock neurons.

## Data Availability

Supplementary material is available online [41].
